# The Hospital Officers' Association

**Published:** 1906-12-29

**Authors:** 


### THE HOSPITAL OFFICERS' ASSOCIATION.

### DO HOSPITALS GIVE EXCESSIVE FREE MEDICAL RELIEF?

A Meeting of the Hospital Officers' Association was held at the New Gaiety Restaurant on Friday, December 21, Mr. P. Michelli, C.M.G., in the chair, when Sir Henry Burdett delivered an address on "Excessive Free Medical Relief; Medical Discontent; and the Remedy for Both."

The Chairman, after expressing his regret at the unavoidable absence of the President of the Association (Mr. Conrad W. Thies) through illness, called on Sir Henry Burdett to deliver his address.

Sir Henry commenced by explaining that he did not intend to go over the same ground as he covered in his speech at the United Hospitals' Conference recently, except so far as it might be necessary to do so with a view to bringing out certain facts. He wished that evening, speaking to them as men who were actively engaged in the administration of hospitals, to try and see whether they could not regard that question of free medical relief as a matter of business, as well as a matter of public duty, and address themselves to the question of what a business consideration of the subject could do to help the hospitals financially and to help the public to obtain efficient medical relief.

### The Result of the United Hospitals' Conference.

Judging by an article which had been published in the *Hospital Gazette*, there appeared to be a distinct misunderstanding as to the objects of and the results arrived at by the United Hospitals' Conference. The writer of that article said that, so far as the proceedings were concerned, the whole matter could be briefly summed up in the words "quite impotent." That was an entire misunderstanding of the result of the Conference. The Conference was the first attempt to bring together the members of the medical profession and the managers of hospitals with a view to trying to devise some method by which they could get into closer relations so as to face together the difficulties which surrounded various hospital questions in the hope that a satisfactory solution of them might be found. It was true that originally the resolution which was proposed contained the expression that the Conference "generally approved" of some thirty or more proposals in a long memorandum, and he as the mover accepted in substitution therefor the words "welcome the further consideration of" those proposals, as he thought that that more nearly expressed the spirit in which the Conference had been called. But that Conference then proceeded to appoint a Standing Committee, with the result that there were now two committees, the Standing Committee, representative of the managers of hospitals, and a Hospitals Committee, representing medical men whom he hoped would arrive at some basis of agreement which would enable the Joint Committee to prepare a programme for the Conference to be held in 1907. He, therefore, hoped the Hospital Officers' Association would agree with him that it was a misunderstanding of the result of the Conference to say that the proceedings could be justly, properly, or wisely described as "quite impotent."

### Free Medical Relief.

What was the actual position of this question of free medical relief? So far as patients were concerned it was manifest that there had been a continuous and ever-growing increase in the numbers admitted to treatment, and that those numbers were altogether beyond the increase which had taken place in the population of the metropolis. Thirty years ago, at the time that Sir Charles Trevelyan read his paper, it was stated that in London the number of patients treated at the hospitals bore the proportion of one to four of the population. Now—in the year 1904—the number of patients returned by the hospitals (in numbers some 2,100,000) bore the proportion of nearly one to two of the population. That growth was far and away beyond the growth in the actual population, and he felt very strongly that it showed that too ready a measure of free medical relief had been granted to all who chose to seek it at the hospitals. Of course, in dealing with those figures, they must bear in mind that they were subject to qualifications from the fact that several patients might have been counted more than once for reasons he had already explained, but, making all modifications or deductions they reasonably could, the fact remained that the actual amount of free medical relief rendered by the hospitals was certainly greater than it ought to be had due regard been paid to the fact that no person should be granted free medical relief at any hospital unless his social circumstances and his medical needs absolutely justified such a course.

In the next place there had been a very considerable increase in the expenditure of the hospitals of the Metropolis in the last ten years, and, whilst their resources had been augumented in that period by the charitable public by something like half a million a year, there was yet a very constant cry for more money.

This brought him to the point he wished to urge very strongly on their consideration that evening, and that was that free medical relief had attained a very high point and had entailed upon the hospital managers the necessity of always going to the charitable public with very strong appeals for still more and larger gifts. If they had a strict regulation of the amount of medical relief to be given, and matters were put on a really business footing, they would be able to arrange their expenditure accordingly, with the result that they would never again have the distressing position in which some hospitals found themselves to-day. This would enable the hospital managers to adopt the system of a yearly budget—a system which was practised in America—under which the expenditure could be adjusted to meet the probable receipts for the year.

### Medical Discontent.

On this point he would not say much that evening. There was undoubtedly solid ground for medical discontent, and it must not be regarded as a matter which affected only the medical profession. It had a much more serious bearing upon the public, as the public had to depend upon the medical profession for treatment in their own homes. He did not believe that there was any extensive abuse of the hospitals in the sense in which the public believed it to exist—namely, that people drove up to the hospitals for treatment in broughams, dressed in sealskins—but there was abuse in the sense that the hospitals were doing in the out-patient departments, and in the casualty departments especially, an immense amount of work which ought to be done, and could be properly and better done, by the Poor Law. The Poor Law at the present time had at their infirmaries excellent accident rooms; and there existed this anomaly, that, under the Poor Law, where the circumstances of the patient warranted it payment was exacted, whilst in voluntary hospitals similar cases were admitted gratuitously. Why he believed many cases could be better treated by the Poor Law than as out-patients at the voluntary hospitals was because the patients could be granted food and nourishment by the Poor Law authorities (which was very often what the patients required more than physic), whilst at the voluntary hospitals they could not.

### The Remedy.

Dealing last of all with what he might call the remedy, he thought it could be found in an alteration of system and method. If he had the authority and power he would be prepared to close the out-patient departments for six months, relying on the casualty departments, strictly administered, to afford all the relief which might actually be required for urgent cases. They would then find, he believed, that the majority of the out-patients would have very little difficulty in providing themselves with all the medical treatment they actually required. In that way they might speedily arrive at a solution of their difficulties. Next he advocated the payment of all the medical staff of the hospitals, as this would give the committees the entire control of the business of the hospitals, and they would be able to institute stricter economies, especially in regard to medical and surgical matters. Then there was the question of the provision of paying wards, to which patients above the humbler class might be admitted—persons whose circumstances and home surroundings did not enable them to have the treatment they required in their own homes. In the United States and in foreign countries there was a system of graded hospital beds. The cases which would correspond to our Poor Law cases were put into the fourth class at a certain rate, which was paid for by the commune or the municipality. The next class, corresponding to the ordinary hospital patient was admitted into the next grade, the third class, where they paid a small but inclusive rate. Above that were two other grades, in the second one of which the patient provided the full cost of his treatment, and in the first the fees benefited the hospital funds. It was an undoubted fact that a system of pay-wards was badly needed in this country. The difficulties of providing it were very great, and might be said to be threefold—first, the question of site, for many of the great hospitals had not room to provide accommodation for the erection of pay-wards; secondly, the question of capital for their erection; and thirdly, the medical difficulty, as in order to work satisfactorily the pay-ward must be distinct from the ordinary hospital, whilst it had to be administered and nursed by the authorities of the general hospital. Some of the paying-system difficulties might be got over by an adequate system of insurance which would provide the necessary cost of hospital care and medical treatment for a certain period during each year for a reasonable annual premium. He had had that subject under consideration for many years, and one of the leading actuaries had gone into the matter, and was of opinion that it was possible for a reasonable payment, as in the case of an accident policy, to provide a policy of insurance which would give the patient so many weeks of hospital care, and also a further time during which he might obtain the benefit of country or sea air.

### The Discussion.

The Chairman said that he had for a long time felt that if abuse existed in the out-patient department it was probably caused by the present mode of administering the Poor-law. There was no doubt in his own mind that there was stored both in the wards of the Poor-law infirmaries and in the Poor-law dispensaries a huge store of clinical material of the highest value to medical science. This material now had to be supplied by the voluntary hospitals, but he could not conceive why we were the only country in the world where the clinical material in the Poor-law infirmaries was lost to science. He did not agree with Sir Henry Burdett as to the payment of the hospitals' medical staff.

The Hon. Sydney Holland said he regretted to again find himself in opposition to Sir Henry Burdett, because he (Mr. Holland) had always frankly admitted that in former years Sir Henry had done good work for the hospitals. Unfortunately of late years Sir Henry had separated himself from the everyday detail work, and had drifted into the position of their critic. It was a great pity that he and Sir Henry, both of whom were workers in the hospital world, should have to oppose each other, as he (Mr. Holland) certainly intended to do. He was independent, he was not dependent on Sir Henry's praise or blame, or the praise or blame of his paper, nor was he dependent on Sir Henry for his position in the hospital world—he was afraid it would not be a very high one if he were. It however devolved on someone to oppose Sir Henry, and therefore it devolved on him.

Now what was Sir Henry's argument? It was a little complicated, but so far as he could follow it, it was this, that in the last ten years the population of the administrative county of London had increased 6 per cent., but because during that time the number of hospital patients had increased 23 per cent. there must be an abuse of hospitals. Admitting that Sir Henry's figures were correct—and on this point he had his own opinion—what did Sir Henry's argument assume? It assumed first that ten years ago we were treating all the patients who ought to have been treated. Sir Henry sterilised hospital treatment at the ten-years-ago figure, and said that the hospital patients should since then only have increased in the same proportion as the population, and that therefore 17 per cent. of the patients now treated are unfit subjects for hospital relief. But what was the actual experience of hospital managers? Why had they had to add so largely to their hospital accommodation in London? It was not for their own amusement, but because the subjects for hospital relief were there and had to be provided for. In his (Mr. Holland's) opinion they were even now not providing poor people with sufficient indoor and outdoor relief. Let them think of the difficulty of getting into the Brompton or the Victoria Park Hospital for Consumption; let them remember that vast South London was without a single hospital—except, of course, Guy's and St. Thomas's—and how could anyone possibly say that there were even enough hospitals in London to-day? The figures too were misleading in this way. The population of London was millions, and an increase of even a million in that population only meant an increase of 20 per cent., whilst an increase of a thousand or two thousand patients meant a huge increase in the percentage of patients, and would give Sir Henry reason to blaspheme again. Then Sir Henry said the hospital committees ought to manage their hospitals on business lines, and, before they started work each year, say, "These will be our responsibilities and this will be our income for the coming year." Was there a secretary present that evening who could tell what the income of his hospital would be next year? ("No, no.") The idea was an absurd one. Then Sir Henry assumed that the increase in the number of patients took place in the out-patient department generally. That was not so; the principal increase was in the special departments—certainly it was so in the hospitals with which he was connected. For instance, out of 86,000 out-patients that they had last year at one of his hospitals, 35,000 were received into the special departments, and only 51,000 were ordinary out-patients. This latter number compared with 52,000 ordinary out-patients treated in 1899. So that it would be seen there had not only not been an increase, but there was actually a decrease in the number of ordinary out-patients. Then Sir Henry assumed that the returns made by hospitals as to the number of patients treated by them were correct. Sir Henry knew as well as any man did that the returns of different hospitals were made out on different bases, and that the same man might be returned twice or even more times as attending the same hospital, and he therefore had no right to take the return made by the hospitals on which to base his views of the patients treated.

In conclusion, Mr. Holland said that the whole question was, Is there abuse of hospitals? Sir Henry said, "Yes, because of the increase in the numbers and proportion"; but he (Mr. Holland) said "No!" and he said so from the experience of daily work at the hospital.

Dr. Hogarth and Mr. Tanner, both medical men, differed from Mr. Holland, and said that the hospitals were abused by people who could afford to pay for medical relief. The latter stated that at a recent meeting of the division of the British Medical Association, of which he was chairman, at which the subject was "Hospital Abuse," numbers of cases of this character, all of which were within the personal knowledge of the medical men present, had been narrated.

Other speakers followed.

Sir Henry Burdett's Reply.

Sir Henry Burdett, in replying, said that Mr. Holland had accused him of abusing the hospitals. For that statement there was absolutely no foundation in fact. His article in the *Times* of two years ago was not an abuse of hospitals; it was a strong defence of them, for he felt, as one who had studied hospital questions for many years, as one who had brought money to the hospitals, as one who was interested in large numbers of people in them, and as one who had done his best, from conviction, to make the voluntary hospitals the best hospitals in the world, that it was necessary that plain facts should be recognised and a word of caution given. He believed that there was an absolute necessity that a halt should be called in the ever-growing volume of free medical relief at our hospitals. Speaking to the workers in hospitals, as he was that evening, he could assure them that they must look that matter fairly and squarely in the face and see that some definite, close, continuous attention was paid to the volume of the relief which each hospital gave. Mr. Holland represented the largest London hospital—one which must be very little abused, if it was abused at all. It was situated in a very poor district, and he (the speaker) did not suppose it would be possible for London to continue without the services the London Hospital rendered to the poor. But from that very circumstance it must not be deemed that the experience of the London Hospital was on all fours with the experience of the other hospitals of the metropolis, and so far as he could judge from a continuous study for more than 30 years of the figures, there was undoubtedly—let them make whatever allowances they might—too much free medical relief given. All he would ask them to do was to be fair, and, before they gave any judgment either as to motive or as to the figures which had been called into question, to study the figures, and study what he had actually written as it appeared in the first chapters of the last two volumes of "Burdett's Hospitals and Charities." When they had done this he was quite content to leave with them the decision as to whether he had abused the hospitals, or whether, in having published those statements, he was not entitled to claim to stand forward as one of the best friends, if not the best friend, that the voluntary hospitals had in this country to-day.

Referring to some of the other points which had been raised, Sir Henry said that as far as the hospital population figures were concerned, he did in his opening address make certain qualifications. Mr. Holland made other qualifications. But after all these figures were the official figures of the hospitals themselves, and they were the figures on which the individual hospitals had based their appeals for money. In this connection he would like to say this, that he knew that attempts were being continuously made to improve the accuracy of the figures given in the hospital reports, but he would urge on hospital officials that wherever possible every figure in the annual report should be closely questioned before it was issued, so that even greater accuracy might be arrived at. He thought, however, that the figures must be reasonably accurate now from the fact that the King's Fund had recently obtained returns from the hospitals of the attendances of the out-patients, and the average attendance of each out-patient had been shown to be 2.6——

The Hon. Sydney Holland: That includes dental patients. The ordinary out-patient's average is 5.

Sir Henry Burdett: The average attendance had been shown to be 2.6. The attendances on this basis made the actual number of patients relieved practically agree with those given in the hospital reports. Mr. Holland's remark showed how difficult the whole question was. If, therefore, every one of his hearers would endeavour to make clear in their reports exactly what the limitations of their figures were and what they actually meant as they understood them, it would be a very great step towards helping those who supported hospitals and those who ventured sometimes to criticise them. Mr. Holland had alluded to the Budget statement, and on this point he (the speaker) must differ from him. Mr. Holland said the hospitals could not make up a budget statement. But it was possible to do so in business, and there was no difficulty in doing it in the hospitals of America. What they did was to make up a statement of probable receipts, expenditure, and work on the conservative basis of an average of two or three years, and that gave them a very good indication of what resources lay before them in the coming year, and of what limitations, as men of business, they would have to place upon the volume of the work done. Such a course should be adopted systematically by every modern hospital.

A vote of thanks to Sir Henry Burdett for his address was carried unanimously, and the proceedings then terminated.

